# A review of the effectiveness of cognitive-behavioral group therapy on the reduction of body image concern in patients with breast cancer

**Published:** 2015

**Authors:** J Faraji, A Mahdavi, E Samkhaniyan, SH Asadi, N Dezhkam

**Affiliations:** * Clinical Psychology, Islamic Azad University Saveh Branch, Iran; ** Payame Noor University, Department of Psychology, Faculty of Psychology and Educational Sciences, University of Payam Noor, Tehran, Iran; *** Health Psychology, South Tehran University; Student Health Psychology, Department of Education and Psychology, Islamic Azad University, Karaj, Iran; **** General Psychology, Islamic Azad University, Roudehen Branch, Iran; ***** Evaluation and Measurement, Islamic Azad University, Central Tehran Branch, Iran

**Keywords:** group therapy, cognitive-behavioral, body image concern, patients with breast cancer

## Abstract

**Objective:** Taking the appropriate psychological actions to boost the mental health of patients with breast cancer is critical. This research was performed with the aim of examining the effectiveness of cognitive-behavioral group therapy on reducing body image concerns in patients with breast cancer.

**Methodology:** TThe method used was quasi-experimental with a pretest-posttest plan and control group. Therefore, 40 patients with breast cancer who had referred to the oncology and radiotherapy department of Imam Hossein Hospital of Tehran were selected by convenience sampling method and organized into two groups: experimental and control group. Both groups were pretested by using demographic and body image concern questionnaires. Then the experimental group received cognitive-behavioral group therapy training for eight sessions and the control group did not receive any intervention. Afterwards, both groups were post-tested, and the data were analyzed by using SPSS software with descriptive and inferential statistics methods.

**Findings:** The findings showed that the cognitive-behavioral group therapy training significantly contributed to the reduction of body image concern in patients with cancer (p < 0.001).

**Conclusions:** It was concluded from this research that cognitive-behavioral group therapy training is an effective strategy to help patients with breast cancer who suffer from the concern about body image due to its high efficiency, especially when it was held in groups, it had low cost, and it was acceptable by the patients.

## Introduction

The first thing every person belonging to any era faces in knowing himself is the physical and bodily aspect. In fact, the physical appearance of individuals makes the first impression of the person by others, and these ideas are also useful on the individual’s attitude toward himself [**1**]. However, the appearance of individuals may be affected due to factors such as some diseases. Among women, cancer is the second leading cause of death, and its most common type is breast cancer. Breast cancer is the uncontrolled development of abnormal cells in different areas of the breast, and often beginning as a painless lump or hardness in the upper and outer of the breast, and in general, it can form anywhere in the breast, including the nipple. Breast cancer, particularly in women, endangers the identity of the patients. It exposes them to problems such as anxiety, depression, hopelessness, and feelings of social isolation, fear of the reaction of the spouse, worries about marriage in case of celibacy, fear of death, and fear of sterility [**2**].

In other words, in diseases that affect people’s physical appearance, body image is a critical factor [**1**]. The term body image is an abstract word and applies to attitudes that persons have toward their bodies as an objective reality [**3**]. The results of a recent research on people who have a functional or physical problem with their body showed that four aspects are assumed for body image that include 1. Comfort (which manifests in the consciousness of patients for their body sensory experiences such as pain) 2. Adequacy (which will manifest in the feeling of change related to the ability of the body to perform activities and actions) 3. Appearance (which is visible on the changes related to what the body looks like) and 4. Predictability (which refers to the stability of the body image over time, especially the balance about defects in actions or reactions) [**4**,**5**].

In addition, this is while the overall body image dissatisfaction has increased during the past 30-40 years [**6**]. Many people, especially women, spend a lot of time, effort, and money to achieve the perfect body image. Women are more challenged with the distortion of body image compared to men, which is most likely due to social messages about women’s roles and expectations, which are often contradictory and confusing. According to Saules KK, Collings AS, Hoodin F, Angelella NE, Alschuler K, Ivezaj V et al. [**7**], the body image is conceptualized based on a multi-faceted structure. In fact, body image is a complex concept that involves biological, psychological, internal, and external social factors. The combinations of cognitive, attitudinal, and behavioral evaluations to body image are better predictors of body image disorders [**8**]. Various researchers have shown that there a positive relationship between physical attractiveness and happiness and success. Therefore, those life events that are associated with significant bodily changes can be useful in the attitudes of people toward their bodies. On the other hand, adverse physical signs have shown a significant negative correlation with life satisfaction [**9**]. Given the high impact of body image on various aspects of the life of the individual, studying the conditions or interventions that can improve the patient’s mental image, are of great importance. 

Cognitive-behavioral therapy is among the therapeutic interventions whose effectiveness was shown in various fields. Cognitive-behavioral therapy is a form of psychotherapy that focuses on how people think about a situation, and in a sense, the emotions of individuals are affected by the way of thinking and helps people understand thoughts, feelings, and attitudes that affect their behavior (Patten, 2012) [**10**]. Cognitive-behavioral therapy is a short-term treatment and during it, which is usually eight to twelve sessions, the person learns how to identify and change the destructive or disturbing effects and thought models that have an adverse impact on his/ her behavior [**11**]. There are many emphases in the cognitive-behavioral approach, such as the concepts operationally expressed, and the treatments, which are empirically validated. Most of the treatment is done based on the approach of “Here and Now”, and it is assumed that the primary objective of the treatment is to help the patient to make desired changes in his/ her life [**12**]. Therefore, during treatment, people learn to control their thoughts, and identify those thoughts that cause feelings and actions, and have an opportunity for the new adaptive learning and making changes outside the clinical scope [**13**]. According to what has been said, it seems that cognitive-behavioral therapy is effective in improving the body image of the patients with breast cancer, therefore, this study was performed to examine the effectiveness of the cognitive-behavioral group therapy on improving the body image concern in patients with breast cancer. 

## Methodology

The study was quasi-experimental with pretest-posttest and control group. The statistical population of the survey included all the patients with breast cancer who referred to the Department of Oncology and Radiotherapy in Imam Hossein Hospital of Tehran from December 22 to March 19 2015. Given that the appropriate sample size in experimental studies is 15 people for each group [**14**], a sample size of 15 patients (n = 15) was selected for each cluster. The inclusion criteria included the following items:

A diagnosis of breast cancer according to the specialist doctors.

An informed consent and the willingness to participate in the research.

The ability to take part in the sessions and collaborate in carrying out assignments.

Willingness to cooperate in completing the tools.

Psychological and physical stability (the absence of prominent physical or psychological signs, which could intervene during the attendance of the sessions).

The age between 20 and 45. 

Also, if the case was treated for a physical, psychological or cognitive disorder or impaired cognitive functions, or in the existence of severe disease symptoms in a way that made it difficult or almost impossible for the patient to participate in the study, the patient was excluded from the survey. Therefore, some were selected from the patients having medical records in the Department of Oncology and Radiotherapy of Imam Hossein Hospital in Tehran, and in the case of consent and having the entry criteria, they were randomly classified into two groups of cognitive-behavioral therapy group and control group.

To conduct the research, we referred to the Department of Oncology and Radiotherapy of Imam Hossein Hospital in Tehran. Then, the sampling of all the patients referring to the hospital was started, and, after making sure they met the entry and exit criteria, 40 people were randomly selected from the real people, as the sample, and grouped into groups of 20. Afterwards, explanations were given to the patients in the study, regarding the treatment sessions, and questionnaires of the study, and in the case of a consent of the person to participate in the study, the person was randomly put into each group of experimental or control group. An informed consent was obtained from the participants in addition to providing explanations about the research and the benefits of participating in such sessions before conducting the study in order to uphold the principles of research as well as to ensure the participants who would attend the sessions. Then, the experimental group received group cognitive-behavioral therapy for eight sessions and the control group did not receive any intervention. In the end, both groups were post-tested. The protocol of the sessions of cognitive-behavioral therapy group is presented in **Table 1**.

The devices used in this study included a demographic sample page and a body image concern inventory. 

Demographic questionnaire: the researchers developed this survey to receive the personal information of the participants. Features such as age, education, and marital status of the participants were asked in the survey. 

Body image concern inventory: a body image anxiety list was developed. This inventory had 19 articles that came with five options - (never to always), which were scored in the range of 1 to 5. The researchers reported the Cronbach’s alpha coefficient to be 93% and the validity coefficient of the inventory through Padua obsessive-compulsive to be 62%, and regarding the eating disorders, the inventory was 40% which was good and satisfactory (P < 0.0001). In Iran, the validity through internal consistency and retesting the inventory were reported to be 69% and 78%, respectively. Also, a significant correlation (61%) was obtained between this inventory and the Coopersmith Self-Esteem scale [**14**].

The SPSS-20 software was used for data analysis. The statistical methods used for data analysis of the research on the level of descriptive statistics were mean, standard deviation, frequency, and frequency percentage indexes, and on inferential statistics, the univariate analysis of covariance model was used.

**Table 1:** Protocol of group training sessions of cognitive-behavioral therapy

Session	Subject
First	Acquaintance of the members of the group, getting familiar with group policy, the introduction of depression, anxiety and stress, and knowing their physical effects
Second	Recognizing negative thoughts, how these thoughts are created, learning how to overcome negative thoughts
Third	Learning how to overcome dichotomous thinking, arbitrary inferences, unbalanced judgment, instant conclusion, mind-reading and false inferences
Fourth	Learning how to overcome extreme generalization, labeling, incorrect terms, exaggerated generalization, absolutism, mental filtering, and feelings of guilt
Fifth	Learning how to overcome exaggeration and understatement, tragicness, disastrousness, dichotomous swiftness, too much attention to negative situations, and personalization
Sixth	Knowing the time of getting angry, controlling anger, and overcoming anger
Seventh	Continuing the training, practicing and carrying out the practices, learning relaxation techniques to use in uncomfortable situations
Eighth	A brief overview of the sessions of treatment and providing feedback to each other, training about how to transfer the findings and lessons learned outside the group

**Findings of the research**

The demographic properties of the sample present in the study are presented in **Table 2**. 

**Table 2:** Demographic characteristics of the subjects

Variable	Group	Frequency	Frequency percentage	Mean and standard deviation
Age	25-30	3	7.5	37.65 ± 5.07
	31-35	13	32.5	
	36-40	8	20	
	41-45	16	40	
Level of education	High school diploma	7	17.5	
	Associate Degree	4	10	
	Bachelor degree	22	55	
	Master degree	7	17.5	
Marital status	Bachelor	21	52.5	
	Married	19	47.5	

**Table 3:** Descriptive statistics of the score of the variables of the research in two groups divided by pretest and posttest

Control		Experimental		Index	Component
Posttest	Pretest	Posttest	Pretest		
60.20	59.70	35.30	60.10	Mean	Body image concern
7.70	7.96	7.03	7.46	Standard deviation	

As shown in **Table 3**, the experimental group’ mean scores of body image concern in the posttest stage decreased compared to the control group.

**Table 4:** Results of Levene test for reviewing the assumption of the consistency of variances for body image concern variable in the posttest

Variable	Stage	F	Degree of freedom 1	Degree of freedom 2	Significance level
Body image concern	Posttest	0.418	1	38	0.522

As shown in **Table 4**, the null hypothesis of the equality of variances of two groups in the variable of body image concern was confirmed. It means that for the body image concern variable, the variances of the two clusters in the population were equal and had no significant difference. Thus, given the compliance with the Levene assumption, the analysis of covariance of the results of the hypothesis of the research was permitted.

**Table 5:** The results of multivariate analysis of covariance to assess the effectiveness of group cognitive-behavioral therapy on the variable of body image concern in the posttest stage

Index	Sum of squares	Degree of freedom	Mean squares	F	Significance level	Squares
Body image concern	6201.101	1	6201.101	113.961	0.001	0.750

According to **Table 5**, as the significance level was (p < 0.001), the hypothesis of the difference of body image concern between the two groups was confirmed. Also, it was stated that 0.75% of the change in the body image concern was because of the independent variable (group training of cognitive-behavioral therapy). Therefore, it was said that the group training of cognitive-behavioral therapy improved the body image concern for patients with breast cancer. 

## Discussion and conclusions

Given the objective of the study, which was to review the effectiveness of cognitive-behavioral group therapy on improving the body image concern in patients with breast cancer, the findings obtained from multivariate covariance analysis showed that the group cognitive-behavioral therapy training had a significant impact on improving the body image concern in patients with breast cancer. This finding was consistent with Abolqasemi A, Jafari I, Rajabi S, Hashemi J, Pezeshk Sh, Garavandi S, Gram K, Mazlumi A, Mazlumi E, Safarzadeh A, Roushan R, Shams JD, Ahmadi-Tahoor M studies [**14**-**18**] in different researches. In explaining his similar findings, Ahmadi-Tahoor (2012) stated that the cognitive method is used in cognitive-behavioral therapy to improve mental distortions and beliefs about body image and cognitive errors. Moreover, it was used to explain how to deal with these cognitions, techniques for dealing with stress, such as relaxation, training in how to regulate excitement, and behavioral techniques such as imaginary and real exposure, to mitigate the negative body image. Furthermore, in this approach other similar cognitive and behavioral techniques were used, which helped the patients gradually confront with parts of their body that caused discomfort by using relaxation techniques, and gradually reducing their tension, pain, and anxiety, and thereby get a sense of satisfaction and completeness. Also, in explaining their similar findings, Safarzadeh, Roushan and Shams (2013) stated that in cognitive-behavioral therapy, the individuals are taught cognitive skills which lead to a better self-perception. In fact, cognitive elements in this treatment can somehow include changes in cognition and behaviors that lead to uncomfortable thoughts or actions.

**Acknowledgement**

The authors would like to thank the proper authorities of Imam Hossein Hospital of Tehran for the assistance. The authors would also like to thank all the participants in this study. 
